# The Effect of an Educational Intervention Program on Allied Health Students’ Knowledge and Attitudes Regarding Organ Donation and Transplantation

**DOI:** 10.3390/nursrep16010015

**Published:** 2026-01-07

**Authors:** Falastine Hamdan, Loai Alfarajat, Rafi Alnjadat, Eshraq Almomani, Mohammad Etoom, Salwa AbuAlrub

**Affiliations:** 1 Nursing Department, Faculty of Nursing, Al-Balqa Applied University, Salt 19117, Jordan; falsteen.hamdan@bau.edu.jo; 2 Applied Health Sciences Department, Irbid University College, Al-Balqa Applied University, Salt 19117, Jordan; rafi.alnjadat@bau.edu.jo (R.A.); esshraq.momani@bau.edu.jo (E.A.); maetoom@bau.edu.jo (M.E.); salwa.abual@bau.edu.jo (S.A.)

**Keywords:** organ donation, attitudes, knowledge, educational program, allied health students

## Abstract

**Background:** A significant shortage of available organs for transplantation persists globally, with insufficient education on organ donation recognized as a key contributing factor. Allied health students, when equipped with accurate knowledge, have the potential to serve as advocates for organ donation, influencing public attitudes through their social networks. Enhancing their understanding may contribute to increased organ donation awareness and acceptance within the broader community. **Methods:** This study employed a quasi-experimental design to examine the effect of an educational intervention program on allied health students’ knowledge and attitudes toward organ donation and transplantation. A total of 150 allied health students were recruited through simple random sampling. Data were collected using a valid and reliable translated self-administered online questionnaire. Participants were divided into intervention and control groups. Descriptive statistics, independent sample *t*-tests, and one-way ANOVA were used for data analysis. **Results:** Following the intervention, the mean score of knowledge and attitudes in the intervention group (*M* = 41.09, *SD* = 2.57) was significantly higher than that in the control group (*M* = 40.29, *SD* = 2.40), with a *t*-value of −3.49 and a *p*-value of <0.001. These results indicate that the educational program had a statistically significant positive effect on participants’ knowledge and attitudes regarding organ donation and transplantation. **Conclusions:** The implementation of the educational intervention significantly improved allied health students’ knowledge and attitudes toward organ donation and transplantation. This suggests that targeted educational programs for future health professionals may be an effective strategy to promote organ donation awareness and address the shortage of organ donors.

## 1. Introduction

Organ donation refers to the act of providing a human organ or tissue from either a living or deceased individual to a recipient in need of transplantation [1]. The replacement of diseased organs or tissues to restore body function is called transplantation [2]. The process of transferring organs from donors to recipients is governed by rigorous standards and regulations [3].

The concept of organ donation has been mentioned in history from the 4th century BC [4]. The pioneering instance of successful organ donation and transplantation occurred in 1954, as reported by [5]. In Jordan, the first organ donation was performed in 1972 [6]. The establishment of the Jordanian Center for Organ Transplantation Directorate (JCOTD) occurred in April 2010, with operationalization following in October 2011. Subsequently, multiple campaigns promoting organ donation have been launched, alongside the implementation of general provisions to regulate the organ donation process [6]. In 2022, there were 157,526 organ donations made globally, 199 of which were performed in Jordan [7], which accounts for 0.001% worldwide. However, Spain is a global leader in donations, with 40.2 donors per million population [8].

The scarcity of organs is a major public health concern [9]. In 2017, 130,000 organ transplants were completed worldwide, accounting for less than 10% of the global need [10]. In the United States, approximately 42,000 organ transplants were performed in 2022, while 104,234 potential recipients were on the waiting list [11]. In Jordan, 500 Jordanians require kidney transplantation each year in order to live a normal life, and another 1800 require corneal transplantation in order to regain their sight [12]. However, only 316 Jordanian organ donors were registered (251 of them were living donors, and 65 were deceased donors) [13]. Hence, a significant disparity exists globally between the supply and demand for organs [14].

Organs that can be donated and transplanted include the kidneys, heart, lungs, liver, and body tissues such as skin, corneas, heart valves, and bones [15]. Globally, the most commonly performed procedure is kidney transplantation [16]. Autografts, which refer to organs or tissues transplanted within the same individual, contrast with allografts, which involve transplants between two distinct human recipients [17]. Allografts can originate from either living donors or cadaveric (brain dead) sources [17]. On the other hand, in certain conditions, such as infectious disease (HIV) and specific cancers (neoplasms), the donation of organs is contraindicated [18].

The end stages of renal, hepatic, pulmonary, and cardiac disorders cause the vital organs to fail [19]. Organ transplantation represents a highly effective therapeutic intervention that significantly enhances the quality of life and prolongs the long-term survival of patients experiencing organ failure [20]. Also, organ transplantation reduces morbidity and mortality rates, allows for medical and social recovery, and lowers medical care costs [21]. It is reported that a person can save up to eight lives if they give all of their organs; if they donate both tissues and organs, they can save 75 more lives [14]. However, several individuals with organ failure die due to the lack of transplantable organs [22]. Approximately 17 humans die each day waiting for organ transplantation [11], and 6000 deaths occur every year among patients awaiting organ transplantation [23].

The field of organ donation is complicated and multifaceted, encompassing medical, legal, ethical, organizational, and social considerations [24]. The global demand for organ transplantation continues to far exceed the available supply capacity [25]. This could be related to the lack of awareness, knowledge, myths, misconceptions regarding organ donation, and improper infrastructure facilities [24,26]. Also, a scarcity of organ donors and an extended waiting list for recipients [27]. There is a significant deficit between patients in need of transplants and donor volunteers [28]. Therefore, enhancing knowledge levels and raising awareness about organ donation and transplantation play a critical role in facilitating the success of Jordan’s organ donation program.

Religious beliefs, income disparities, and ethical and cultural considerations have been identified as primary obstacles to organ donation [16,29]. It was reported that religion and culture affect decision-making processes [30]. In addition, age, gender, marital status, and income could influence the willingness to donate organs [31]. Furthermore, previous studies revealed that there is a significant impact of educational intervention programs on students’ knowledge and attitudes toward organ donation and transplantation [32,33]. Hence, to increase the availability of possible organ donors, efforts towards identifying factors affecting organ donation and conducting educational programs need to be intensified.

People are hesitant to donate their organs [4]. Previous studies reported that previous interactions with health care providers, family issues, and body integrity as potential factors that could influence organ donation [34]. Family members have indicated a reluctance to donate organs because they are afraid the surgery would harm their health [25]. Furthermore, ineffective rules and regulations, mistrust towards organ donation, poor advertising by relevant organizations, and deceased donor programs that are still in development could hinder organ donation [25,31]. Furthermore, Alizadeh and Takasi, 2024 [35] conducted a systematic review study to assess the level of knowledge of nursing students regarding organ donations and the factors that affected this knowledge. Their study targeted all studies published at Scopus, PubMed, Web of Science, and Persian databases. The studies included were only cross-sectional studies applied to nursing students and all recent studies until the 12 August 2023. The number of nursing students, 2048, was represented across the twelve studies conducted. The researchers concluded that nursing students lacked knowledge of organ donation. They recommended investigating factors that affected knowledge and attitudes toward organ donation by conducting further interventional and comparative research. This result is consistent with the present study, which supports the necessity for conducting our study. In Jordan, obstacles to establishing a deceased-donor program include paucity of public knowledge and awareness regarding brain death; scarcity of trained transplant personnel, such as surgeons, physicians, coordinators, and nurses; scarcity of competent support units; and lack of faith and confidence in the success of complicated procedures such as liver and heart transplants [6]. Therefore, it is crucial to assess the factors that influence organ donation and transplantation in Jordan.

The majority of university students have strong educational backgrounds, originate from various regions, are proficient in using technology, and have a wealth of information and experience. As a result, they are a significant and unique group in society [16]. Previous research has clearly shown that knowledge and attitudes are the key elements that improve organ donation rates [36,37,38,39]. Therefore, in order to enhance the effectiveness of organ donation and transplantation, it is imperative to augment public awareness and deepen knowledge in this critical domain [28].

The attitudes of healthcare providers can influence people’s commitment to donate organs for transplantation [39]. The knowledge and attitudes of healthcare workers towards organ donation and transplantation have a positive influence on organ donation rates [28]. Healthcare providers are the initial point of contact for the potential donor’s family; they can increase the organ donation rate by raising awareness and removing barriers through consultation, education, and counseling [40]. They can provide precise information and cultivate a positive attitude towards organ donation among prospective donors and their families, motivating them to enroll for an organ donor card and contribute their organs later after death [41]. Conversely, inaccurate information about organ donation and transplantation may negatively influence an individual’s decision to become an organ donor [42]. Given that allied health students serve as future healthcare providers and initial points of contact for potential donors’ families, they play a pivotal role in advocating for organ donation and transplantation. Notably, prior research has highlighted gaps in allied health students’ understanding of organ donation processes [12]. Furthermore, these students exhibit deficiencies in knowledge and attitudes toward organ donation and transplantation [43,44]. Consequently, there is a pressing need to enhance allied health students’ knowledge and attitudes regarding organ donation and transplantation, providing them with opportunities for self-assessment and educational interventions.

### Study Aims

- Examine the effect of an educational intervention program on allied health students’ knowledge and attitudes regarding organ donation and transplantation.
- Identify the relationship between sociodemographic characteristics and knowledge and attitudes regarding organ donation and transplantation.

## 2. Methodology

### 2.1. Design and Setting

This study adopted a quasi-experimental two-group pre-test/post-test design, conducted at a governmental university in Jordan. The research focused on allied health students aged 18 years and older. The intervention program was implemented from 19 November 2023 to 30 December 2023.

Although participants were assigned to either the intervention or control group using a coin-flipping method, no formal procedures for allocation concealment or randomization sequence generation were implemented. Therefore, the study is appropriately classified as quasi-experimental. No trial registration or blinding was performed.

### 2.2. Participants

A probability random sampling method was used to select participants from the list of enrolled allied health students obtained from the university registration office. The inclusion criteria were:

➢ Age 18 years or older
➢ Enrollment as an allied health student
➢ Ability to read and understand Arabic
➢ Access to the internet
➢ No prior education on organ donation and transplantation
➢ Willingness and ability to participate

Participants were then non-randomly assigned to either the intervention or control group using a basic coin-flipping method without allocation concealment or a pre-determined randomization sequence, which limits the internal validity and precludes classification as a randomized controlled trial.

The required sample size was estimated using G*Power 3.1.9.7 software for a two-tailed independent samples *t*-test, with the following parameters: medium effect size (Cohen’s d = 0.5), significance level (α) = 0.05, and statistical power (1 − β) = 0.80. This yielded a minimum required sample size of 64 participants per group, or 128 total.

To account for an anticipated attrition rate of 15%, the sample size was increased. The adjusted sample size was calculated as:

128 ÷ (1 − 0.15) ≈ 151

Thus, the target was set at 75 participants per group (total n = 150).

Given the actual sample size of 75 per group, the post hoc power analysis (α = 0.05, n = 75 per group, d = 0.5) shows an achieved power of approximately 0.88 [45,46].

### 2.3. Ethical Considerations

Ethical approval was obtained from the Institutional Review Board (IRB) at Al-Balqa Applied University (Approval ID: 26/3/2/2505) to collect data. Approval for the study to commence was granted on 19 November 2023, Informed consent was sought from each participant through an online consent form. The study’s purpose and anticipated outcomes were explicitly communicated to the participants. They were voluntarily invited to participate, with the right to decline or withdraw at any point without penalty. Confidentiality of their responses was assured.

### 2.4. Data Collection Tools

The study tool consisted of a self-administered questionnaire. The questionnaire was hosted through Google Forms, and it consisted of three parts. The first part of the questionnaire was the demographic data (age, gender, marital status, academic program, academic year, grade point average (GPA), residency, smoking, mother’s education, and father’s education).

The second part was a validated tool related to knowledge and attitudes regarding organ donation and transplantation, which was adapted from the literature [45,46,47,48]. The knowledge part consisted of 13 questions, while the attitudes part consisted of 10 questions. The tool used a dichotomous scale (Yes/No) to measure the participants’ responses. Each “Yes” response received a score of 2, while each “No” response received a score of 1. Questions 6, 9, 10, 21, 22, and 23 were reverse-scored, as the correct responses for these questions were “No”. The total scores were calculated by adding up the individual scores. Higher scores indicated higher levels of knowledge and more positive attitudes regarding organ donation [46].

In the present study, the questionnaire underwent translation into Arabic language, followed by a back-translation process facilitated by two professionals (The Arabic version tool is available upon reasonable request). The content validity was assessed by five nursing experts, revealing excellent content validity (CVI = 0.83). Additionally, a pilot study was conducted to evaluate the internal consistency of the translated tool. The pilot study involved 10 participants (who were subsequently excluded from the final analysis). The Cronbach’s alpha of the tool was 0.82. The Cronbach’s alpha for the knowledge subscale (13 items) was 0.79, and for the attitudes subscale (10 items) it was 0.84, both of which indicate acceptable to good internal consistency.

### 2.5. Data Collection Procedure

**Figure 1:**
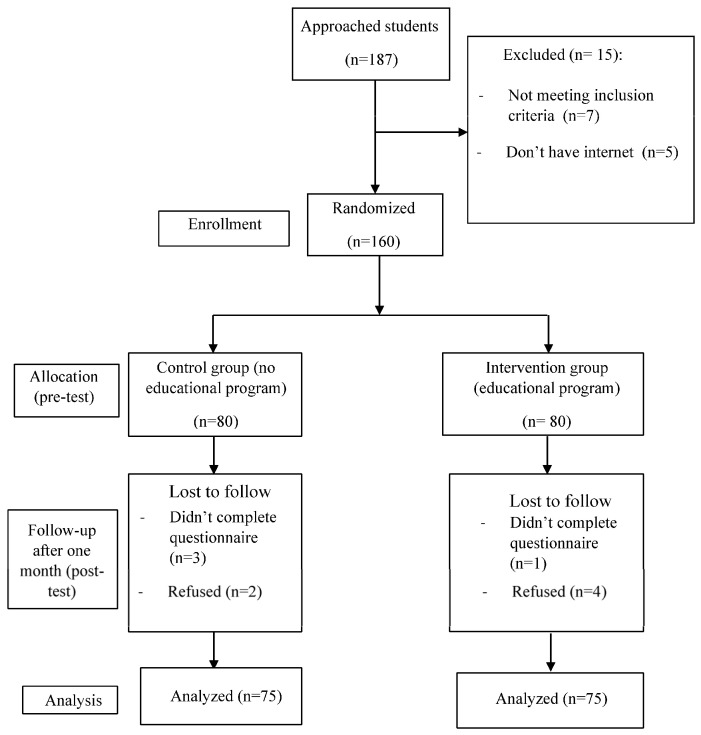
Consolidated standards of reporting trials (CONSORT) guidelines flow chart of participation in the organ donation and transplantation educational interventional program study.

Google Forms were used to collect the data, which was then extracted as an Excel file. The Google Form link contained the study questionnaire and was shared by the researchers to study participants in a specific Microsoft Teams class designed for the current study. CONSORT’s [49] explanation and elaboration guideline for randomized trials was used in this study (Figure 1).

The study group (I) received a detailed education about organ donation and transplantation, while the control group (II) did not receive any intervention. For ethical reasons, the control group received the same intervention after the post-test data collection.

Following the approval of participation in the study, and approving the online consent form, participants completed a short demographic data form and a knowledge and attitudes questionnaire before joining the educational intervention. The pre-test data collection occurred over 5 min.

The intervention was delivered to participants by specialized doctors and instructions from the Jordanian Organ Donation Association. The educational content was multifaceted, which included definitions, types, organs that can be donated, contraindication for donation, statistics regarding organ donation, real stories about organ donations, and legal, cultural, and religious views of donation. The researchers held one teaching session for participants in the experimental group, which lasted around 4 h. Lectures and discussions were conducted during the session. Also, the teaching session included specific images and videos concerning organ donation and transplantation.

Post-test data collection began one month after the intervention. We had the students complete the online questionnaire to assess their knowledge and attitudes regarding organ donation and transplantation, and whether they had maintained, enhanced, or deteriorated.

### 2.6. Data Analysis

The Statistical Package for Social Sciences (SPSS, version 27) for Windows was used for data analysis. Descriptive statistics were utilized to describe and review the demographic data according to the level of measurement. Inferential statistics, including *t*-test and one-way ANOVA, were used to assess the knowledge difference between groups and to determine differences in knowledge and attitudes regarding organ donation and transplantation.

## 3. Results

A total of 187 students were approached to participate, due to which the final count was eventually affected by different reasons, such as the lack of internet access, not meeting the inclusion criteria, and loss to follow-up (see Figure 1). In the end, only 150 students completed the full program (pre/post-tests), which were equally assigned to the control group (n = 75) and the intervention group (n = 75). The mean age of participating students was (*M* = 20.9, *SD* = 2.46). Also, more than two thirds of participants were female (n = 113, 75.3%), single (n = 138, 92%), in the second year of study (n = 121, 80.7%), and nonsmokers (n = 121, 80.7%).

The mean academic percentage of participating students was (*M* = 74.6, *SD* = 8). Also, more than half of the participants were studying nursing (n = 83, 55.3%). In addition, the highest percentage of participants lived in town (n = 143, 95.3%). Furthermore, most study participants reported that the study level of their mother and father was high-school level (n = 70, 46.7%, n = 81, 54%, respectively). Table 1 represents the participants’ demographic characteristics.

An independent sample *t*-test was employed to assess the differences between the participants in the control and intervention groups prior to and after the implementation of the educational program. The intervention group (*M* = 41.09, *SD* = 2.57) had a significantly higher mean score of knowledge and attitudes regarding organ donation and transplantation compared to that of the control group (*M* = 40.29, *SD* = 2.40); *t* = −3.49, *p* < 0.001) (Table 2).

A bivariate Spearman correlation was conducted to assess the relationship between demographic variables (age and GPA) and participants’ knowledge and attitudes in the intervention group. The result revealed that there is no association between age and GPA and knowledge and attitudes (Table 3).

An independent sample *t*-test was conducted to compare the differences in knowledge and attitudes level among study participants based on demographic variables (gender, residency, and smoking). The results revealed that the differences in knowledge and attitudes level among study participants are not statistically significant across all these demographic variables (Table 4).

A one-way ANOVA was conducted to compare the differences in knowledge and attitudes level among study participants based on demographic variables (marital status, academic program, academic level, mother’s and father’s education). The results revealed that the differences in knowledge and attitudes level among study participants are not statistically significant across all these demographic variables (Table 5).

## 4. Discussion

Several descriptive studies in the literature that assessed knowledge and attitudes toward organ donation among health-related personnel emphasized a vital need for educational programs [50,51]. Such programs would enhance knowledge and attitudes toward this topic. Therefore, this study aimed to examine the effect of an educational intervention program on allied health students’ knowledge and attitudes regarding organ donation and transplantation.

The current study showed that the intervention group had a significantly higher mean score of knowledge and attitudes concerning organ donation and transplantation in comparison with the control group. This suggests that the intervention program had a beneficial impact on this subject matter. The results were consistent with earlier research conducted on medical students [52,53]. The possible explanation of this result is that most students expressed enthusiasm in learning about organ donation and the program efficiency in delivering that information. In contrast, only one study in the literature reviewed showed that fine art students demonstrated no statistical significance with regards to both an increase in their positive attitudes toward organ donation and inclination to possess a donor card after peer learning [52]. There was no indication that the efficiency of peer learning affected the attitudes of the fine arts students toward organ donation and obtaining an organ donor card. Researchers attributed this result to the fact that fine art students did not spend enough time in their faculty to obtain the optimum benefits from peer learning because they had an individual study style. The authors from that study noted the absence of descriptive or interventional research regarding the attitudes of fine arts students towards organ donation. The majority of interventional research on organ donation has been conducted with students in the field of health sciences. The study was the first to focus on improving the attitudes of fine arts students regarding organ donation [52].

In the present study, lecture and discussion sessions included videos on organ donation and transplantation delivered by a physician and an instructor from the Jordanian Organ Donation Association. These videos were directed to allied health students and had a positive impact on their knowledge and attitudes toward organ donation. There have been several learning strategies utilized across studies to improve knowledge and attitudes toward organ donation. For example, one study conducted by [54] emphasized that a structured training program that included audiovisual lectures and materials, such as pamphlets, enhanced the knowledge and perceptions of nursing students toward organ donation. This study evaluated knowledge and perception level before, immediately after, and one month following the intervention. The scores reached their peak immediately after training, then declined after one month of training but remained higher than the results before training. In addition, this result is consistent with the recommendations of Bertocchi et al. (2025) [55] who investigated nursing students’ knowledge and attitudes toward organ donation and transplantation among nursing students in Italy. They conducted a cross-sectional study for this purpose. Researchers recommended the importance of virtual active learning methodologies as a strategy to increase awareness of donation in tandem with face-to-face active learning methodologies. They also recommended designing strategies to increase health profession students’ education, awareness, and adherence to donation, which supports the necessity for conducting our study.

Another study implemented a peer learning strategy by educating nursing students’ leaders about organ donation, who then conducted peer learning to their colleagues who were included in the study. This strategy was effective in significantly improving attitudes of nursing students toward organ donation [52]. Moreover, ref. [54] developed an interactive educational strategy of both web-based and classroom lectures, which included videos, discussions, and quizzes. The post-intervention test indicated that nursing students in the experimental group had a higher level of knowledge and a higher intention to register for organ donation than students in the control group.

Education intervention targeting students contributed to improving organ donation indirectly. They would discuss the topic with their families, relatives, and friends. In one of the studies, an educational intervention directed to 1144 high school students in India resulted in an increase in students’ willingness to register for organ donation from 66.9% to 80.9%. Also, the percentage of students who discussed the topic with their family members after the education session was 82.4%. Of this group, 4.9% of the students stated that one or more of their family members registered to donate their organs after death [56].

The current study’s results revealed no statistically significant difference in the mean score of knowledge and attitudes level regarding organ donation and transplantation among participants in the intervention group, based on the participants’ demographic variables. This result could be related to an unbalanced sample size in each study group, which might lead to difficulty in detecting a significant variation among study participants. Also, this could be explained by the homogeneity and culture of the population, as the highest percentage of participants lived in towns. Thus, they could have similar levels of knowledge and attitudes due to their shared background, religion, and social norms. This result was consistent with an Indian study that targeted 100 undergraduate university students to explore the effect of a structured teaching program in the students’ knowledge and attitudes. It found that there was no relationship between students’ knowledge or attitudes level and demographic variables, such as age, sex, religion, and residency setting [57].

Educational intervention is an essential approach in promoting organ donation knowledge and attitudes. Individuals who have a high level of knowledge are more likely to register as organ donors [58]. Education interventions should be directed to high school students, undergraduate students in colleges and universities, healthcare professionals, and the public as a whole. Integrating the organ donation concept in schools and university curricula, implementing classroom education, and training student leaders to educate their peers are effective methods to enhance knowledge and attitudes [56,59]. This approach was also emphasized as a recommendation by Alwesmi et al. (2023) [60] who applied a cross-sectional comparative design to investigate the knowledge, attitudes, and factors affecting nursing students’ knowledge and attitudes toward organ donation. A study was conducted among Princess Nourha bint Abdurrahman University nursing students. The results revealed a low score of knowledge (6 out of 15), which contradicted the high level of positive attitude toward organ donation. The researchers specified a number of factors that contributed to this low level of knowledge: willingness to donate, donor registration, and donation medals based on their number and type of donated organs, academic level, academic performance, and knowledge level. The researchers recommended increasing these students’ knowledge by including education regarding organ donation and transplantation practices in nursing curricula. An additional recommendation emphasized the importance of receiving additional systematic, structured knowledge to encourage more nursing students to become registered organ donors and increase the number of potential donors among future healthcare providers.

Public awareness regarding organ donation can be increased by establishing national campaigns using social media, TV, pamphlets containing answers for frequent questions, and online education tools [61]. Ref. [13] conducted a quantitative survey in Jordan among 500 participants. This survey found that more than 50% of the participants use the internet and social media (Facebook and Snapchat) as the main sources of knowledge about organ donation. In comparison, healthcare providers (16%) and school education (24%) had the lowest percentage as sources of knowledge.

Further research is needed in Jordan to identify the long-term influence of educational intervention on allied health students’ knowledge and attitudes regarding organ donation, the impact of students’ knowledge and attitudes level on organ donation, and to explore the barriers that could hinder organ donation. We recommend conducting communication skills training for healthcare professionals, especially those who are in direct contact with patients and families in emergencies and intensive care units, to effectively convey information to prospective patients and their relatives about organ donation and transplantation [61]. According to [62], a qualitative study, involving 18 Intensive Care Units (ICU) Nurses, showed a lack of nurses’ knowledge regarding organ donation evaluation criteria, policies, and protocols. In addition to poor communication skills, there were barriers to ICU nurses encouraging patients and their families to donate organs [1].

Also, healthcare systems should implement structured training programs to educate healthcare providers and patients about organ donation. For example, Europe established the European Training Program on Organ Donation and the European Donor Hospital Education Program [15].

## 5. Strengths and Limitations

The strengths of this present study included the utilization of a quasi-experimental method with a pre- and post-test for both the intervention and control groups. Also, the study used a probability random sampling method to assign participants to intervention and control groups, which decreased bias. More than half of the participants were nursing students. These individuals were aspiring nurses who would serve as the primary communicators to patients and their families, potentially educating them about organ donation. In addition, the study employed a variety of instructional techniques such as lectures, discussions, visual aids, and videos to educate participants on the subject matter.

Nevertheless, this study did have limitations. The sample was drawn exclusively from a single university, and the demographic characteristics of the students were not representative of the wider population. Therefore, the findings may not be applicable to all groups of participants in the research. In the future, collecting demographic information, such as age, gender, and cultural and religious background, will enable us to conduct targeted studies on students’ knowledge and attitudes about organ donation.

## 6. Future Perspectives

While current evidence strongly supports the effectiveness of educational programs in enhancing organ donation awareness and attitudes among allied health students, several areas warrant further exploration to optimize these interventions. Future research should investigate the comparative efficacy of varying durations and intensities of educational interventions. For instance, longitudinal studies examining the sustained impact of single-session versus multi-session programs could provide insights into optimal program duration and reinforcement strategies.

Additionally, there is a need to evaluate alternative pedagogical approaches, including the integration of simulation-based learning, digital modules, and peer-led discussions. Comparative studies assessing the relative benefits of these methods could inform the design of more engaging and impactful curricula. Exploring the role of personalized learning pathways that account for students’ baseline knowledge, cultural backgrounds, and professional goals may also yield more tailored and effective interventions.

Furthermore, future studies should consider broader implementation outcomes such as behavioral intent, actual registration rates, and long-term retention of knowledge and attitudes. Multicenter trials and cross-cultural comparisons would enhance the generalizability of findings and support the development of context-specific educational strategies. By addressing these research gaps, future work can inform evidence-based, scalable models for organ donation education that align with the evolving needs of healthcare education and practice.

## 7. Conclusions

In conclusion, the evidence overwhelmingly supports the notion that educational programs play a crucial role in promoting organ donation among allied health students. These programs serve as invaluable platforms for raising awareness, disseminating accurate information, and fostering positive attitudes towards organ donation. By equipping future healthcare professionals with the knowledge and skills necessary to engage in meaningful discussions about donation, such initiatives not only empower individuals to make informed decisions but also cultivate a culture of compassion and altruism within the healthcare community.

Furthermore, educational programs have been shown to address misconceptions and dispel myths surrounding organ donation, thereby alleviating fears and uncertainties among students. Through interactive learning experiences and case studies, these programs offer opportunities for students to explore ethical dilemmas, cultural considerations, and legal frameworks associated with donation, ultimately preparing them to navigate complex scenarios with sensitivity and professionalism.

Moreover, the impact of educational interventions extends beyond individual attitudes and behaviors, contributing to broader societal shifts in perception and acceptance of organ donation. By fostering a generation of allied healthcare students who champion the cause of donation, these programs have the potential to increase donation rates, save lives, and improve outcomes for patients in need of life-saving transplants.

## Figures and Tables

**Table 1:** Participants’ demographic characteristics.

	Intervention Group (n = 75)		Control Group (n = 75)		Both Groups (n = 150)	
Variable	*M* (*SD*)	*N* (%)	*M* (*SD*)	*N* (%)	*M* (*SD*)	*N* (%)
**Age**	21.1 (2.89)		20.8 (1.94)		20.9 (2.46)	
**Gender**						
- Male		19 (25.3)		18 (24)		37 (24.7)
- Female		56 (74.7)		57 (76.0)		113 (75.3)
**Marital status**						
- Single		69 (92)				138 (92)
- Married		3 (4)		69 (92)		9 (6)
- Divorced		2 (2.7)		6 (8)		2 (1.3)
- Widow		1(1.3)				1 (0.7)
**Academic program**						
- Pharmacy		21 (28)		11 (14.7)		32 (21.3)
- Nursing		41 (54.7)		42 (56)		83 (55.3)
- Midwifery		13 (17.3)		22 (29.3)		35 (23.3)
**Academic year**						
- First year		5 (6.7)		8 (10.7)		13 (8.7)
- Second year		65 (86.7)		56 (74.7)		121 (80.7)
- Third year		5 (6.7%)		11 (14.7)		16 (10.7)
**GPA**	77.8 (74.6)		69.8 (8.0)		73.8 (53)	
**Residency**						
- Town		73 (97.3)		70 (93.3)		143 (95.3)
- Rural		2 (2.3%)		5 (6.7)		7 (4.7)
**Smoking**						
- Yes		13 (17.3)		16 (21.3)		29 (19.3)
- No		62 (82.7)		59 (78.7)		121 (80.7)
**Mother education**						
- Illiterate		6 (8)		2 (2.7)		8 (5.3)
- Primary		7 (9.3)		4 (5.3)		11 (7.3)
- High school		35 (46.7)		35 (46.7)		70 (46.7)
- Diploma		16 (21.3)		22 (29.3)		38 (25.3)
- BSC		9 (12)		11 (14.7)		20 (13.3)
- Master’s degree or higher		2 (2.7)		1 (1.3)		3 (2)
**Father Education**						
- Illiterate		5 (6.7)		6 (8)		11 (7.3)
- Primary		7 (9.3)		4 (5.3)		11 (7.3)
- High school		38 (50.7)		43 (57.3)		81 (54)
- Diploma		8 (10.7)		12 (16)		20 (13.3)
- BSC		10 (13.3)		8 (10.7)		18 (12)
- Master’s degree or higher		7 (9.3)		2 (2.7)		9 (6)

*N* = 150; *M* = Mean; *SD* = Standard Deviation.

**Table 2:** Independent *t*-test for participants’ knowledge and attitudes scores between the intervention group and the control group.

Test Score	Intervention Group (n = 75) *M* (*SD*)	Control Group (n = 75) *M* (*SD*)	*t*
**Pre-test knowledge and attitudes score**	38.61 (3.14)	40.21 (2.40)	1.96
**Post-test knowledge and attitudes score**	41.09 (2.57)	40.29 (2.40)	−3.49 ***

*M* = Mean; *SD* = Standard Deviation. *** *p* ≤ 0.001 (2-tailed).

**Table 3:** Relationship between age and GPA and participants’ knowledge and attitudes.

		Age	GPA
**Knowledge and attitudes** **score** **Intervention group (n = 75)**	***r_s_***	0.011	0.073

**Table 4:** Differences in knowledge and attitudes level based on demographic variable.

Variable	*N* (%)	Knowledge and Attitudes Score *M* (*SD*)	Test Statistic
**Gender**			*t* = −0.079
- Male	19 (25.3%)	41.05 (2.27)	
- Female	56 (74.7%)	41.10 (2.69)	
**Residency**			*t* = 0.052
- Town	73 (97.3%)	41.09 (2.56)	
- Rural	2 (2.3%)	41 (4.24)	
**Smoking**			*t* = −0.445
- Yes	13 (17.3%)	41.38 (2.98)	
- No	62 (82.7%)	41.03 (2.50)	

Intervention group, *N* = 75; *M* = Mean; *SD* = Standard Deviation.

**Table 5:** Differences in knowledge and attitudes level based on demographic variable.

Variable	*N* (%)	Knowledge and Attitudes Score *M* (*SD*)	Test Statistic
**Marital status**			*F* = 1.271
- Single	69 (92%)	41.15 (2.61)	
- Married	3 (4%)	39 (1.0)	
- Divorced	2 (2.7%)	43 (1.41)	
- Widow	1 (1.3%)	39	
**Academic program**			*F* = 0.193
- Pharmacy	21 (28%)	40.80 (2.99)	
- Nursing	41 (24.7%)	41.24 (2.44)	
- Midwifery	13 (17.3%)	41.07 (2.43)	
**Academic year**			*F* = 1.729
- First year	5 (6.7%)	43 (1)	
- Second year	65 (86.7%)	41 (2.5)	
- Third year	5 (6.7%)	40.2 (3.3)	
**Mother education**			*F* = 0.417
- Illiterate	6 (8%)	41.1 (2.63)	
- Primary	7 (9.3%)	41.4 (1.90)	
- Secondary	35 (46.7%)	41 (2.62)	
- Diploma	16 (21.3%)	40.7 (3.10)	
- BSC	9 (12%)	41.1 (2.26)	
- Master’s degree or higher	2 (2.7%)	43 (0.70)	
**Father Education**			*F* = 0.213
- Illiterate	5 (6.7%)	41 (3.39)	
- Primary	7 (9.3%)	41.5 (1.71)	
- Secondary	38 (50.7%)	41 (2.86)	
- Diploma	8 (10.7%)	41.6 (1.99)	
- BSC	10 (13.3%)	40.5 (2.22)	
- Master’s degree or higher	7 (9.3%)	41 (2.57)	

Intervention group, *N* = 75; *M* = Mean; *SD* = Standard Deviation.

## Data Availability

The datasets generated by the current study are not publicly available due to privacy reasons, but are available from the corresponding author on reasonable request.

## Public Involvement Statement

This research involved students as active partners.

## Guidelines and Standards Statement

This manuscript was drafted against the Consolidated Standards of Reporting Trials (CONSORT) 2010 statement: extension to social and psychological interventions (CONSORT-SPI 2014) for randomized research studies.
